# Supplementary material for: Seminoma and Embryonal Carcinoma Footprints Identified by Analysis of Integrated Genome-Wide Epigenetic and Expression Profiles of Germ Cell Cancer Cell Lines
Source: PLoS One. 2014 Jun 2;9(6):e98330. doi: 10.1371/journal.pone.0098330 (PMC4041891; doi:10.1371/journal.pone.0098330)
Supplement: File S1 — ZIP file containing DMRforPairs output for significant regions. Please start from the html files. (ZIP) [file pone.0098330.s008.zip › figures/2852.pdf]

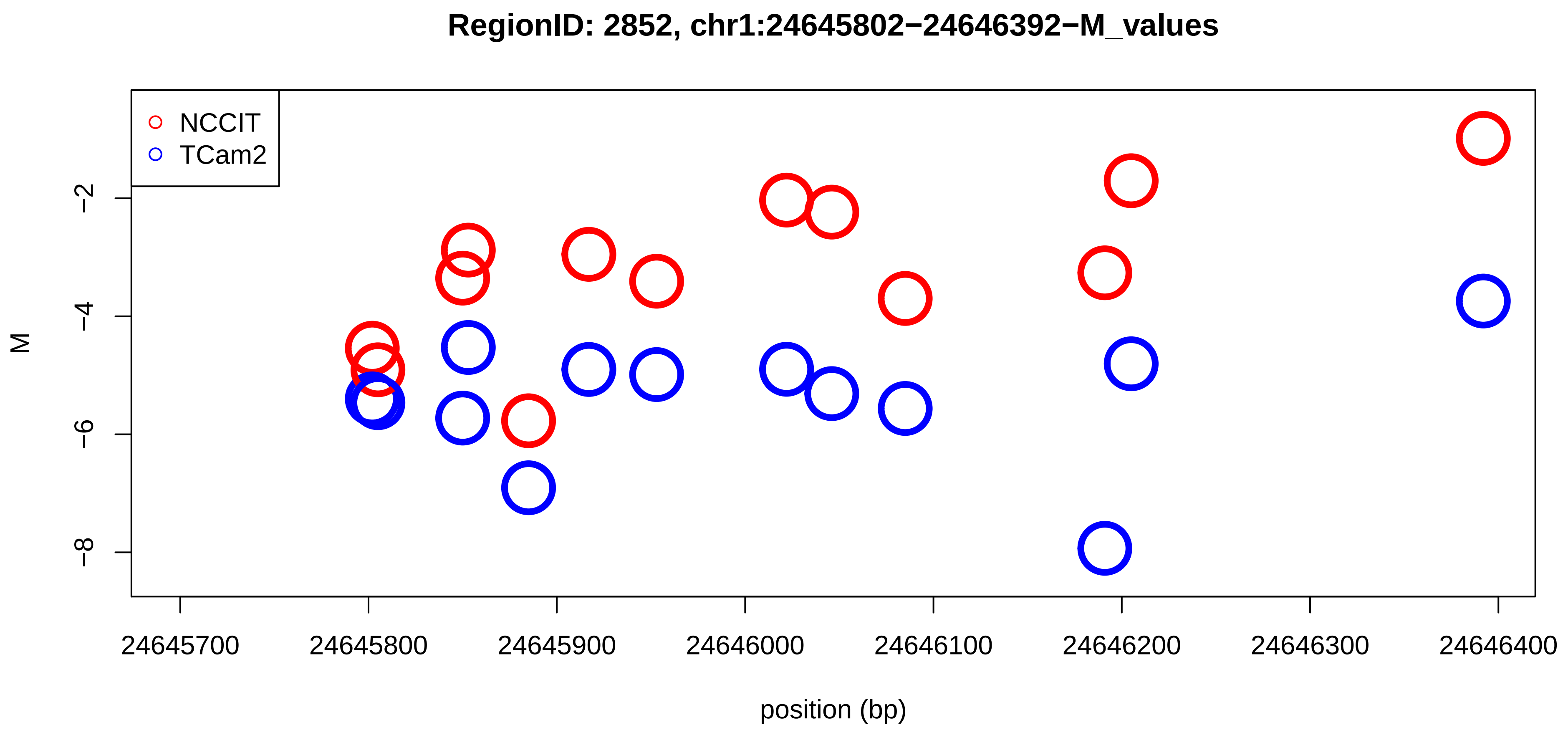
RegionID: 2852, chr1:24645802−24646392−M_values
NCCIT
TCam2
M
−2
−4
−6
−8
24645700
24645800
24645900
24646000
24646100
24646200
24646300
24646400
position (bp)

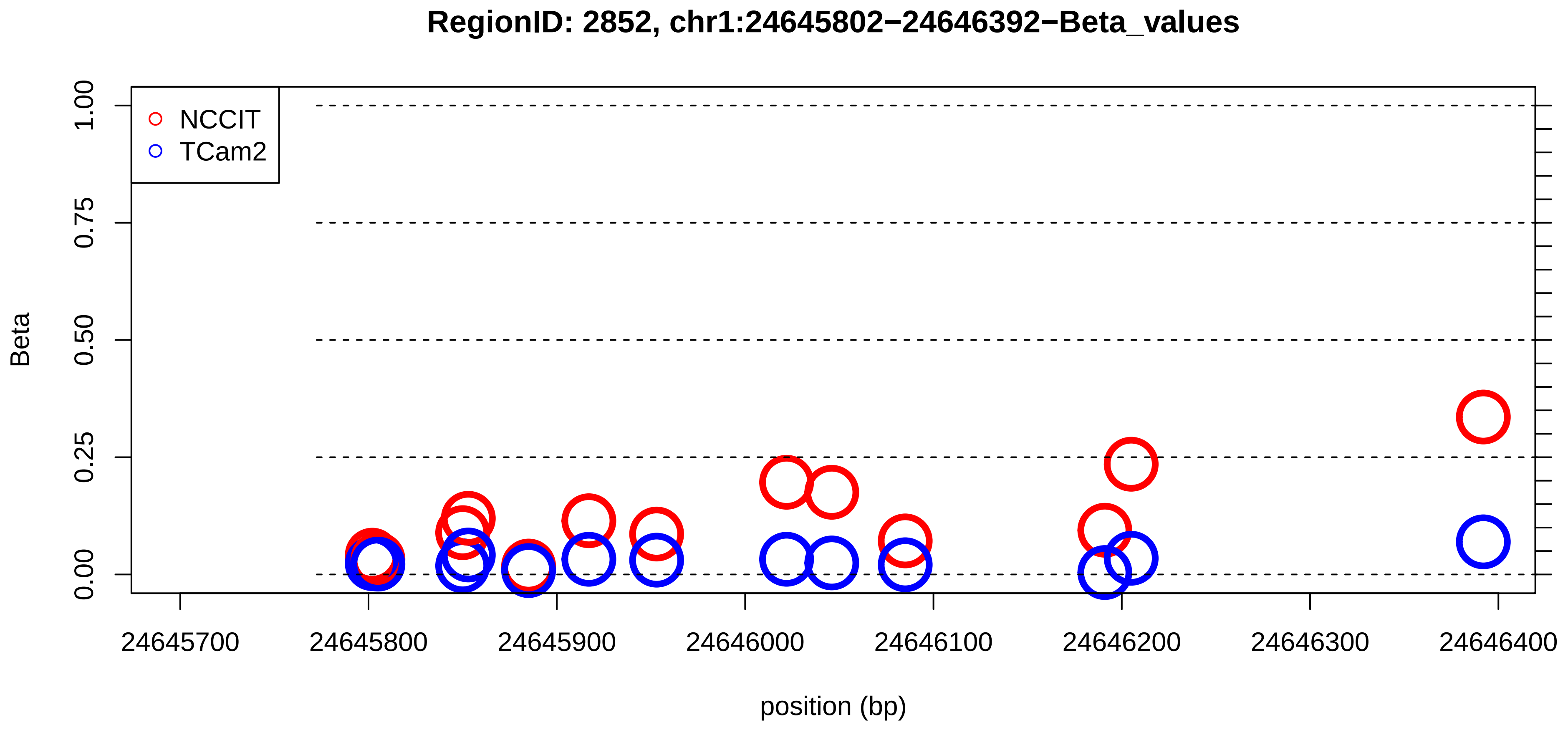
RegionID: 2852, chr1:24645802−24646392−Beta_values
NCCIT
TCam2
Beta
0.00
0.25
0.50
0.75
1.00
24645700
24645800
24645900
24646000
24646100
24646200
24646300
24646400
position (bp)
